# The role of health system governance in strengthening the rural health insurance system in China

**DOI:** 10.1186/s12939-017-0542-x

**Published:** 2017-05-23

**Authors:** Beibei Yuan, Weiyan Jian, Li He, Bingyu Wang, Dina Balabanova

**Affiliations:** 1 China Center for Health Development Studies, Peking University, Beijing, China; 2 Department of Health Policy and Management, School of Public Health, Peking University, 38 Xueyuan Road, , Haidian District PO Box 505, Beijing, 100191 China; 3 London School of Hygiene and Tropical Medicine, London, UK

**Keywords:** Health system, Governance, China, Rural areas, Health financing, Health insurance

## Abstract

**Background:**

Systems of governance play a key role in the operation and performance of health systems. In the past six decades, China has made great advances in strengthening its health system, most notably in establishing a health insurance system that enables residents of rural areas to achieve access to essential services. Although there have been several studies of rural health insurance schemes, these have focused on coverage and service utilization, while much less attention has been given to the role of governance in designing and implementing these schemes.

**Methods:**

Information from publications and policy documents relevant to the development of two rural health insurance policies in China was obtained, analysed, and synthesise. 92 documents on CMS (Cooperative Medical Scheme) or NCMS (New Rural Cooperative Medical Scheme) from four databases searched were included. Data extraction and synthesis of the information were guided by a framework that drew on that developed by the WHO to describe health system governance and leadership.

**Results:**

We identified a series of governance practices that were supportive of progress, including the prioritisation by the central government of health system development and certain health policies within overall national development; strong government commitment combined with a hierarchal administrative system; clear policy goals coupled with the ability for local government to adopt policy measures that take account of local conditions; and the accumulation and use of the evidence generated from local practices. However these good practices were not seen in all governance domains. For example, poor collaboration between different government departments was shown to be a considerable challenge that undermined the operation of the insurance schemes.

**Conclusions:**

China’s success in achieving scale up of CMS and NCMS has attracted considerable interest in many low and middle income countries (LMICs), especially with regard to the schemes’ designs, coverage, and funding mechanisms. However, this study demonstrates that health systems governance may be critical to enable the development and operation of such schemes. Given that many LMICs are expanding health financing system to cover populations in rural areas or the informal sectors, we argue that strengthening specific practices in each governance domain could inform the adaptation of these schemes to other settings.

## Background

There is increasing evidence that health system governance is critical to health systems operation and overall performance [1, 2]. Experience from a range of countries suggests that governance has been a driver of success in countries, achieving major advances in health and access to care compared to others at a similar level of wealth [3]. Health system governance is shaped by the wider governance framework within the country; however there are indications that governance in the health systems is a strong independent determinant of health system effectiveness and its capacity to achieve its goals [1]. Despite recognising the centrality of governance within health systems, there is a considerable lack of clarity as to what it means vis-a-vis other core functions (‘building blocks’) of the health system. Whether governance is one of the key ‘building blocks’ or a cross-cutting function underlying all other health system functions is a matter for debate.

Importantly, the precise mechanisms through which good or poor governance affects health systems goals (health, financial protection, responsiveness and improved efficiency) are still insufficiently understood. According to the WHO, the concept of leadership and governance “involves ensuring strategic policy frameworks exist and are combined with effective oversight, coalition building, regulation, attention to system design and accountability” [4, 5]. This concept focuses on the stewardship role of the government in governing and managing health care, and coordinating other actors engaged in health policy formulation and its implementation. Other definitions have looked beyond the role of the government, defining governance as the activities through which a society organizes itself to achieve health of populations [6, 7]. Under an effectively governed health system, the policy goals that are set are more likely to be translated into policies and activities that bring benefits to the population, including otherwise excluded groups; policies are more likely to be well designed; and the governments are better able to plan, manage, regulate and implement them [2].

In the last six decades, China has made significant advances in health system strengthening and improving health outcomes, despite suffering setbacks including political and socio-economic crises. The development of a health insurance system for rural residents has been an important means of expanding access to essential care. The new rural cooperative medical scheme (NCMS) in China was established in 2003, as a flagship policy aimed at rural populations. It expanded rapidly, with coverage increasing from 9.5% to 98.9% of rural residents in 2003-2013 [8]. Achievements include extending insurance coverage and improving access to health care for rural residents in China—a group that has previously had only limited access to often rudimentary health care—and alleviating the financial burden associated with seeking care [9]. The NCMS builds on earlier experience with the national cooperative medical system (CMS), which was created in the 1950s and expanded to cover 90% of villages in less than 20 years [10]. These successive schemes were seen as major contributors to the process of health system strengthening, supplementing and enabling other key policies such as the three-tier service delivery model (an extensive and integrated network consisting of health service facilities at county, township and village level linking primary health care with higher levels of care) [11]. Despite differences in their structures and processes, the two schemes have many similarities in terms of policy formulation and implementation, especially featuring the policy innovation and rapid scale up. Though there has been considerable attention by researchers to the impact of the rural health insurance schemes on coverage and service utilization [12, 13], the role of governance—within the schemes, and of the broader health system—in designing and implementing these initiatives, has received limited attention. Specifically, the governance processes underpinning the formulation and implementation of the two health insurance schemes, as well as the pathways through which governance has promoted positive outcomes are insufficiently explored. This represents a considerable gap in knowledge, hampering efforts to understand how the schemes have achieved many of their objectives and what governance features may be needed for their implementation. Filling this gap will provide evidence that is relevant to other low and middle income countries (LMICs) considering similar strategies.

In this paper, we explore these two policies through the lens of governance: a) we identify the governance policies and practices that have underpinned and shaped these two initiatives and enabled them to benefit from innovation and rapid implementation nationally, b) we assess the extent to which these governance practices conformed to the criteria for good governance as conceptualised by WHO and others, with relevance to governance structures, processes and relationships that promote health system strengthening, c) we then explain what common features of governance, operationalised through a specific set of functions, may have supported the implementation and performance of the two key national policies (CMS and NCMS), and identifying lessons for low and middle income countries (LMICs).

## Methods

We reviewed information from publications on the development of two rural health insurance policies in China. The information was identified, extracted, arranged and analysed according to the WHO framework for health system governance (Table 1) [5, 6], which includes six dimensions: policy guidance and vision, system design, regulation and management capacity, accountability and transparency, intelligence and oversight, collaboration and coalition building. Within each function, supportive (*good*) governance should manifest as [5]: 1) a clear positioning and health policy vision, 2) explicit and systematic policy design and implementation strategy, 3) appropriately designed regulations or incentives aligned with policy objectives, 4) effective supervision and accountability mechanisms established to support implementation, 5) evidence-informed policy design and scientific oversight on implementation of health policy, and 6) coordination and collaboration among different sectors.

**Table 1:** Health system governance functions and the specific attributes to be analysed for each domain

Governance domain	Features of good governance [5, 6]	Specific questions
Policy guidance and vision	Formulating sector strategies and also specific technical policies; defining goals, directions and spending priorities across services; identifying the roles of public, private and voluntary actors and the role of civil society.	Any long term health plans/development goals/any documents? Role of “Health” in overall country development? Any other technical plans or strategies for this policy? Are goals and priorities clearly defined in the policy strategies? Are the policy strategies and designs comprehensive? Who initiated the policy (e.g. central government, departments and its level, individual, or media)? Why initiated the policy (e.g. problems targeted, or high level will)?
System design	Ensuring a fit between strategy and structure and reducing duplication and fragmentation.	Is the specific health policy related to overall health or national development plan? To what extend the system structure responds to what needs to happen in this policy? Design of interventions and policies to deliver services Are the duplication and fragmentation reduced?
Regulation and management capacity	Designing regulations and incentives and ensuring they are fairly enforced.	How the policy is operationalized (e.g. regulation, contract or legal)? How to ensure the different levels of government accountable to the policy aims (e.g. supervision, promotion of leader)? How administrative structure is organized and contribute to the enforcement of policy (e.g. duties allocation, reporting to higher authority) What regulations or incentive to ensure the implementation? How to ensure the management capacity of different levels of governments (e.g. selection, training, supervision)
Accountable and transparent	Ensuring all health system actors are held publicly accountable. Transparency is required to achieve real accountability.	How to ensure the health policy be responsive the priority of health problems at that time? How the different levels of governments adopt policies based on the needs of local residents? Is the performance of health authorities or health providers transparent to the public? Is the finance situation of health authorities or health providers or health insurance fund transparent to the public?
Intelligence and oversight	Ensuring generation, analysis and use of intelligence on trends and differentials in inputs, service access, coverage, safety; on responsiveness, financial protection and health outcomes; on the effects of policies and reforms; on the political environment and opportunities for action; and on policy options.	What is the basis for policy design and contents? Is evidence underpinning policy design? How are pilots evaluated? How are the local experience and lessons formulated and disseminated? What monitoring and evaluation systems are in place? How the monitoring and evaluation contribute to policy?
Collaboration and coalition building	Across sectors in government and with actors outside government, including civil society, to influence action on key determinants of health and access to health services; to generate support for public policies, and to keep the different parts connected - so called ‘joined up government.	How actors work across different sectors on design and implementation of policy? How are different actors mobilized to contribute to health activities? How is the collaboration with civil society organizations or other (outside the formal health sector)? How are the different levels of government collaborating?

### Search strategy

First, we reviewed the literature systematically, including four electronic databases (PubMed, Proquest Dissertation & Theses Database, China National Knowledge Infrastructure (CHKD-CNKI) and Chinese Medicine Premier (Wanfang Data) containing publications in English or Chinese without limitations on the publication date. The websites of the China Health and Family Planning Committee, WHO, and the World Bank were also searched. The search strategy and terms in English and Chinese are listed in Table 2. Historical policy documents were obtained from the archives of the China Health and Family Planning Committee and other ministries holding relevant information. We also obtained advice from experts in health system strengthening and rural health care in China to identify additional relevant research papers, Ph.D. dissertations, reports, and policy and administrative documents.

**Table 2:** Search strategy

Databases searched	Search terms
PubMed (Search date: 8 March 2016)	#1:politics[MH] OR governance[TIAB]OR “policy making”[TIAB] OR “policy-making”[TIAB] OR “policymaker”[TIAB] OR “policy makers”[TIAB] OR “policy-maker”[TIAB] OR “policy-makers”[TIAB] OR decision-maker[TIAB] OR decision-makers[TIAB] OR decentralization [TIAB] OR decentralized[TIAB] OR recentralization[TIAB] OR centralization[TIAB] OR administrator[TIAB] OR administrators[TIAB] OR government[TIAB] OR governments[TIAB] OR regulation[TIAB] OR regulations[TIAB] OR stakeholder[TIAB] OR stakeholders[TIAB] OR responsiveness[TIAB] OR accountability[TIAB] #2: health[TIAB] #3: China[MH] OR China[TIAB] OR CMS[TIAB] OR NCMS[TIAB] OR NRCMS[TIAB] OR “New Rural Cooperative Medical System”[TIAB] OR “health system reform” [TIAB] OR “healthcare reform” [TIAB]
Proquest (Search date: 8 March 2016)	((ti(politics OR governance OR "policy making" OR "policy-making" OR "policy maker" OR "policy makers" OR "policy-maker" OR "policy-makers" OR decisionmaker OR "decision making" OR "decision makings" OR decisionmakers OR decentralization OR decentralisation OR decentralized OR decentralised OR recentralization OR recentralisation OR centralization OR centralisation OR administrator OR administrators OR government OR governments OR regulation OR regulations OR stakeholder OR stakeholders OR responsiveness OR accountability OR equity OR inequity OR inequities OR leadership) AND (ti(CMS OR NCMS OR NRCMS OR "Cooperative Medical System" OR "New Rural Cooperative Medical System" OR "health insurance" OR "health system reform" OR "healthcare reform" OR "Patriotic Health Campaign") OR ti(health AND reform))) OR (ab(politics OR governance OR "policy making" OR "policy-making" OR "policy maker" OR "policy makers" OR "policy-maker" OR "policy-makers" OR decisionmaker OR "decision making" OR "decision makings" OR decisionmakers OR decentralization OR decentralisation OR decentralized OR decentralised OR recentralization OR recentralisation OR centralization OR centralisation OR administrator OR administrators OR government OR governments OR regulation OR regulations OR stakeholder OR stakeholders OR responsiveness OR accountability OR equity OR inequity OR inequities OR leadership) AND (ab(CMS OR NCMS OR NRCMS OR "Cooperative Medical System" OR "New Rural Cooperative Medical System" OR "health insurance" OR "health system reform" OR "healthcare reform" OR "Patriotic Health Campaign") OR ab(health AND reform)))) AND (ti(China) OR ab(China) OR diskw(China) OR su(China) OR au(China) OR sch(China))
China National Knowledge Infrastructure (CHKD-CNKI) (Search date: 8 March 2016)	(主题 = 治理 + 管理 + 分权 + 集权 + 政府 + 规定 + 规则 + 规划 + 利益相关者 + ((设计 + 执行 + 制定)NEAR (方案 + 政策 + 制度))) AND (主题 = 医疗 + 健康 + 卫生 + 合作医疗 + 新农合 + 新医改)
Chinese Medicine Premier (Wanfang Data) (Search date: 8 March 2016)	(题名:(“治理”+”管理”+”设计”+”执行”+”制定”+”分权” + "方案”+”政策”+”政治”+”政府”+”规则”+”利益相关者”))*(题名:(”合作医疗”+”新农合”+”新型农村合作医疗”+”爱国卫生”+”新医改”+”医疗卫生体制改革”+”卫生体系”+”卫生系统”))

### Inclusion criteria

We aimed to include all studies analysing the governance backgrounds of two policies, NCMS and CMS, but very few studies focused primarily on governance aspects of NCMS or CMS. To obtain sufficient information related to the study objectives, we included all articles describing or analysing the detailed process of designing and implementing NCMS and CMS, and then extracted information related to health system governance. The judgement of relevance to health system governance was based on WHO’s definition on governance and its functions (Table 1) [4, 5]. The leadership and governance of health systems adopted in this study refers to role of the government in guiding and overseeing the health system as a whole, and its relation to other actors in all activities related to health. These were applied using a framework comprising six key functions common to all health systems [4.5] (Table 1). Given that the study did not seek to quantify outcomes, but to ensure that all relevant information relevant to the role of governance, even if implicit, is included, the study included not only peer-reviewed papers but also policy documents, commentaries, viewpoints, project reports and policy documents. There were no restrictions on study design and methods in order to capture comprehensively all sources providing information relevant to governance factors or practices. We did not assess risk of bias of the studies included as our goal was not to assess the impact of governance and all publications were appraised in terms of significance, level of detail and relevance to the research questions.

Two of the authors (LH and BW) independently screened the abstracts and titles, and discussed disagreements with the lead author to achieve consensus. The lead author (BY) screened all the full texts and another co-author (WJ) checked the all full texts in order to ensure no important documents were missed. Based on these criteria, we screened 9313 titles and abstracts, found from both databases and references tracing, and retrieved 729 potentially relevant publications. We then examined the full texts of these papers for relevance and scope of information related to any governance dimensions. Finally, 92 papers or documents on CMS or NCMS containing information closely related to the study objectives were selected for data extraction and analysis. The materials we included were published or issued between 1960 and 2016.

### Data extraction and synthesis

Data extraction and analysis was guided by a conceptual framework for functions of health system governance (Table 1) drawing on the WHO’s framework on health system governance and leadership and Siddiqi’s framework for assessing health system governance [1, 4, 5]. Though there is no blueprint for effective governance and leadership that is universally applicable, the assumption underlying the framework was that certain government behaviours and practices, categorised within each of the six key domains of governance, are associated with well-functioning health systems.

Each of these governance domains was then operationalised in specific questions (sub-domains) by a multi-disciplinary international expert group collaborating on a lager project to synthesise the experience of China in health system development and lessons for other countries undergoing similar developments. This involved an initial face-to-face workshop, followed by virtual interaction to refine the framework after the initial stages of analysis. For each domain and each question under these domains, the governance-related policies and practices affecting the design and operation of the two insurance schemes (NCMS and CMS) were extracted and described.

The criteria for supportive or good governance were also drawn from the WHO’ analysis on the features of governance arrangements in the well-developed and performing health systems. Good governance is a fluid concept, often referred to but rarely explicitly defined. The framework of Siddiqi [1] and UNDP [14] emphasised relational principles of governance including: inclusion of and valuing the views of different stakeholders and seeking consensus, in addition to strategic vision and other conventional attributes. The WHO model implicitly defines good health system governance as the presence of progressive policies and actions in each governance domain under the oversight of the government acting legitimately on behalf of the population, while their absence is associated with governance failure [4].

We analysed and synthesised the extracted data using a framework synthesis approach [15, 16]. The rationale for using this synthesis method is that given the large amount of textual data extracted from primary studies, it represents a highly-structured approach to organising and analysing data. Thus, emerging governance issues related to the formulation and implementation of the two health insurance schemes (NCMS and CMS) were categorized and matched against each domain and sub-domain of the framework, allowing new attributes to emerge. The attributes were then synthesised and reorganised hierarchically, for example to identify related and divergent attributes, and governance factors related to an overarching governance domain. Some of the governance attributes in the frameworks were not supported by evidence. Two senior health system researchers with specialist knowledge in this area, and two policymakers who were involved in the process of the design and implementation of the CMS or NCMS, were asked to suggest additional sources relevant to the newly emerging themes and to validate the analysis, drawing on their appraisal of the studies. This was required due to the specific characteristics of governance in China, in the health sector and beyond, where the lack of evidence on particular governance-related aspects could be a significant finding in itself. Finally, practices exhibiting characteristics of good governance were identified and synthesised.

## Findings

We first briefly describe the development and characteristics of two rural health insurance policies (CMS and NCMS), summarised from the literature and policy documents and using the conventional functions of a health insurance scheme. The governance practices underpinning these two policies in each governance domain are then analysed against the criteria for supportive health system governance.

### The development of health insurance system in rural China

After the People’s Republic of China was established in 1949, rural residents had to pay out-of-pocket to obtain care, and there was no consistent, nationally-agreed strategy for how to reduce the financial burden on the population [10]. With the formation and rapid development of the collective economy (the collective ownership of land and other property) in rural areas, the collective fund in each commune started to expand its finance pool to include health care. In 1955, the earliest form of a Cooperative Medical Scheme (CMS) appeared in Gaoping County, Shanxi Province [17]. Its coverage increased rapidly after 1955, from 10% of villages in 1958 to 46% of villages in 1962 [18]. Between 1962 and 1967, further development of the cooperative medical scheme stopped and coverage declined markedly, coinciding with a slowdown in development of collective economic [10]. Since 1969, the coverage of cooperative medical scheme restarted, grew rapidly and achieved its peak, whereby 94.4% of village residents were covered by the CMS in 1976 [19].

After 1978, CMS coverage started to decline, mainly because its economic and structural foundations started to change. The economic system in rural areas started to shift towards a household-responsibility system. For example, land previously collectively owned was transferred to being operated by families under the contract with collectives, which reduced the incentive for households to contribute to a community fund intended for CMS. Between the 1980s and the late 1990s, many pilots projects continued, seeking to re-establish the CMS, while there was also a number of studies on how to design the health financing system in rural China [20]. These pilots were conducted by at different levels of government, by officials, researchers, or residents, consistent with a trend to increasing decentralisation. Though most of these pilots and studies confirmed that the cooperative insurance system was superior to private health insurance or user fees, these did not lead to new initiatives to extent insurance coverage in rural China. The CMS was increasingly marginalised; by 1998 only 6.5% of rural residents in China were covered by cooperative schemes [21].

Given that, by the late 1990s, more than 90% of rural residents lacked health insurance coverage, the large burden of untreated illnesses contributed to poverty in rural areas. The situation became very serious by the end of the 1990s: a study of 114 poverty-stricken counties from 1993 to 2000 [10] found that family bankruptcies due to medical expenses accounted for a third of rural poverty. The World Health Organization report 2000, ranked China’s health system at among the worst in terms of fairness of health care financing [22]. The resulting publicity, and subsequent debates raised the affordability of care high on the policy agenda; health financing for rural residents started to be perceived as a serious need by the policy makers [23]. In a parallel development, accelerated economic growth since 1978 increased resources available at different levels of government, and provided the financial basis for the rebuilding of the CMS [10]. Consequently, in 2002, the central government (national level government) of China published a strategy setting out the objectives and parameters of the new insurance scheme [24]. Since then, the NCMS coverage started to grow rapidly. By 2008, 98.17% of rural village counties had established NCMS, and 91.05% of rural residents were covered by the new health insurance scheme; by 2013, the coverage of rural populations was nearly universal, at 98.9% [8].

### The contents of cooperative medical scheme in its different stages

Table 3 compares the features of the two insurance schemes during the different historical stages.

**Table 3:** Characteristics of the cooperative medical scheme over time

	1955–1978 CMS	1979–1996 Collapse period	2003-present NCMS
Fund collection	• Voluntary enrolment • Public welfare fund from agricultural cooperatives • Flat-raged premium from enrolees • Revenue of village clinics	Only few areas still had traditional CMS, and some researches or government policy pilots applied other kinds of health insurance in few areas. In most areas of rural China, no any health insurance system.	• Voluntary enrolment • Subsidy from different levels of government • Flat-rated premium from enrolees
Risk pooling	• Pooled at the village brigade level • In few cases, pooled at township level		• Pooled at county level • In some areas, pooled at municipality level
Benefit package	• Based on the fund level, firstly coverage preventive and outpatient services in village clinics; • Some areas partly covered referred hospital outpatient visits and referred hospitalization. • Provider payment is not clear.		• Covering both outpatient and inpatient services in different level of health care facilities (with different co-payment levels) • Catastrophic diseases are also partly covered. • Provider payment methods include fee-for-service, capitation and case-based payments

#### Fund collection

For both CMS and NCMS, the eligible populations are those registered as rural residents in China, both employed and non-employed [25]. In both schemes, enrolment of rural residents is voluntary. Under the traditional CMS, that was in place from 1949 to 1978, funds were collected from public sources, such as from agricultural cooperatives, revenues from village clinics, and premiums from rural residents [10]. The premiums collected from rural residents were flat rate, for example in Masheng County the premium for each person was ¥ 1.5 to 2 (about $0.21 to $0.29 at 2016 exchange rate) per year in 1966 [26]. From 1979 to 2002, the existing CMS was financed from different sources, including from the collective funds generated by the village community, household premiums, or subsidies from county and town level governments [27]. Since 2003, when the NCMS was established, it has been funded from subsidies by different levels of government and individual contributions [24]. In NCMS the premiums collected from rural residents were also flat rated, and have increased from ¥10 ($1.45) per year in 2003 to on average ¥490 ($14.2) in 2015 [25].

#### Risk pooling

In most areas the CMS funds were pooled at the village level (the lowest administrative level in China is village, followed by township, county, municipal, principal and country level, as the highest level), and only in a few cases the pools expanded to include the township level [10]. In contrast, the NCMS risk pooling was at the county level [24] (there were 2852 rural counties in 2012, with an average population of 300,000 in one county) [25].

#### Benefit package

The benefit package of CMS included preventive health services, free consultation at the village clinic, free or partly covered drugs at the village clinic and, in a few areas, the CMS also partly covered referred state-owned hospital visits and hospitalization [27]. The NCMS benefit package was more comprehensive, covering outpatient and inpatient services in different levels of state-owned health care facilities (including village clinic, township health centres, county hospital and tertiary hospitals in municipal and principal levels), as well as catastrophic diseases with different co-payment levels [13]. Fee-for-service was the main mechanism for paying health care providers applied by the NCMS, however in recent years, some areas started to pilot capitation and case-based payments [28, 29].

### Governance factors underpinning the design and implementation process of CMS and NCMS

This section synthesises the study findings related to governance aspects and supportive factors relevant to the implementation and operation of CMS and NCMS. These are structured under the six key governance domains following the conceptual framework underpinning this study (Table 4).

**Table 4:** Assessing health system governance underpinning CMS and NCMS

Governance domain	Specific criteria in each domain	CMS	NCMS
Policy guidance and vision	Long term health plans/development goals/strategies; Role of this health policy in overall country development; Policy initiated from populations’ problems; Technical plans or strategies for this policy; Goals and priorities clearly defined in the policy strategies; Policy strategies and designs define the roles of different actors;	There was clear health sector development strategy, but no formal long-term health sector development plan; Establishment of the importance position for CMS in national rule on collective economy development; Initiated from local practices and needs of rural residents; Several official documents were issued by central government to direct local governments to design and implement CMS; Economic and production development was the final aims and this policy was regarded as the important contributor to health and economic development; The policy design guidance documents included the roles of different actors but the contents were not detailed;	A clear vision for health system development was illustrated. But no formal long-term health sector development plan; The development of a health insurance system in rural China was added to the five-year national development plan; Initiated by government but based on social problems; Official documents provided technical plans and guidance for this policy design and implementation; Improving health status and reducing financial burden are the explicitly defined as the goals of this policy; The policy design guidance documents defined the roles of different actors in detail;
System design	Specific health policy is related to overall health or national development plan; Health system structure responds to what needs to happen in this policy; Duplication and fragmentation are avoided and reduced?	Related to national health development strategy; Applying existing health system resources and fit into existing structure; Making use of existing agricultural production cooperatives to manage their CMS funds;	Related to national vision for health system development; Forming an organizational structure to support the NCMS fund management and policy operation; New administration department was built inside the health administration department;
Regulation and management capacity	Appropriate system or measures to ensure the policy to be operationalized (e.g. regulation, contract, legal or incentives); System or measures to ensure the different levels of government accountable to the policy aims; System or measures to ensure the management capacity of different levels of governments;	Issuing regulations and rules to implement by top-down way; Top-down way: Supervision by and reporting to higher authority; Training and learning management experience from typical areas;	Issuing regulations and rules to implement by top-down way; Top-down way and incentives for local government; Training for local government officers and NCMS managers;
Accountable and transparent	System or measures to ensure different levels of governments to adopt policies based on the needs of local residents? The performance of health authorities or health providers is transparent to the public; The finance situation of health authorities or health providers or health insurance fund is transparent to the public;	It was encouraged that the local authorities adopted the CMS design based on local situation; No documents provided relevant information on this specific criteria; In some areas, there were relevant requirement on transparency, but some information implicitly reflected the transparency was not implemented well;	The central government only imposed a basic requirement on policy content, and local governments have rights to design each specific NCMS contents; No documents provided relevant information on this specific criteria; The transparency on the NCMS fund income, expenditures, and reimbursement situation was compulsory;
Intelligence and oversight	Evidence basis is available for policy design and contents; Monitoring and evaluation systems are in place and contribute to policy;	No evidences support at the beginning, but the expansion of policy was based on evidences from typical areas, for which decision makers conducted field investigation; No documents show there was any monitoring on policy implementation;	Academic researches provided evidences for policy planning and design; Pilot and evaluation were required before the scale up of policy to over the country, regular monitoring from superior government and regular reporting NCMS operation situation, and large amount of researches and evaluations on NCMS happened together with the development of NCMS;
Collaboration and coalition building	Different government departments work together on design and implementation of policy; Different social actors outside government contribute to the design and implementation of policy;	No documents show how different government departments were collaborated in CMS; No documents show how actors outside government were mobilized and collaborated in CMS;	Different government departments were involved of process of NCMS initiation and design; Academic research institutions took important roles in the process of NCMS design and implementation;

#### Policy guidance and vision

WHO’ framework of health system governance envisages the presence of clear vision of policy aims and priorities for development, accompanied by explicit guidance on how to plan and design policy [4].

## CMS

In 1951, the third year after the founding of the People’s Republic of China, the central government formulated an overall health sector development strategy: prevention-oriented, utilization of both traditional Chinese and western medicine, combined health work and mass mobilization to ensure accessibility of health services to most of the Chinese population, with priority given to workers, peasants and soldiers [30]. Under this strategy, health sector development in rural areas became a priority for decision makers, which could explain why, although the central government initially had no nationally-agreed policy for health insurance system for rural residents, evidence of the achievements of the CMS in some areas encouraged it to promote the rapid expansion of CMS to the whole country. It was reported that, in 1955, when the prototype CMS scheme was implemented in Gaoping County, the central government sent the vice-minister of the Ministry of Health to visit the county and investigate its operation [31]. The priority given to this scheme across different levels of government was apparent from the publication of numerous regulations to enable its wider implementation. In June 1956, just one year after the first CMS appeared, the First National People’s Congress passed “Advanced Agricultural Production Cooperatives Demonstration Rules” which stipulated that the collective economy should take responsibility for health care of rural residents [10].

The government also sought to provide technical guidance to the lower levels of government on policy design. The guidance was in the form of central government issuing official documents in a top-down manner, but the guidance was brief and summarized the typical policy design of the scheme in particular areas. For example, the key guidance document was the “Report on the National Health Work Conference in Jishan County” and its appendix “Opinions on People’s Communes Health Work” issued in 1960, and this guidance was based on CMS experience in Jishan County and other areas that were quick to implement the scheme [10]. By analysing the contents of these documents, it was found that the guidance tried to define the roles of different actors: the individual and commune having responsibility in financing CMS; the residents, health workers and local government officers having right in supervising the fund of CMS; and different levels of health providers in charge of providing health care [27]. This served to demonstrate who should be responsible to design and implement the CMS.

“*The design of Collective Health Care System (CMS in Qishan County): commune members pay a certain amount of health care fee one year, and they could just pay consultation fee or drug fee when they seek for health care. The commune should subsidise for this system……. Under the leadership of the commune, the health workers, village committee and residents’ representatives should form a management committee of Collective Health Care System. And this committee is in charge of formulating the specific management regulations.*” (From the policy document “Opinions on People’s Communes Health Work”, 1960) [27].

## NCMS

Since the introduction of a market economy in China after 1978, economic development has become a core priority for government and other actors at sub-national level. Health system development was not seen as a priority at that time. There was a constant tension between safeguarding people’s health and pursuing economic growth, with the latter dominating the activities of many health care providers. The traditional CMS started to collapse in most areas of China and no alternative financial protection schemes replace them. This meant that rural residents experienced a heavy financial burden from ill health, with people complaining that it is “hard and expensive to see a doctor” and the cost of seeking care gradually became a serious social problem [32]. When policy makers eventually realised the seriousness of this problem, from the end of 1990s [28], central government again began to focus their attention on the development of the health system. In 1997, the central government issued “Decision of Health Reform and Development” which explicitly stated the vision on health system development, namely that population health must be an important goal of national development, and public finance support for the health system must be secured. While simultaneously recognising other existing health system problems, government took the development of NCMS as the start of the reform, and from 2003 NCMS became a priority policy specially promoted by the government.

Some government practices reveal the priority given to the NCMS within the country development agenda. Firstly, the development of a health insurance system in rural China was added to the national development plan. In the ninth Five-Year National Economic and Social Development Plan (issued in 1996), the goal regarding the cooperative medical system was quite specific “*to expand coverage of the cooperative medical system to 50% until 2000s.*” Then, in October 2002, the central government issued the document “Decision on Further Strengthening of Rural Health Work”, and confirmed the public subsidy that would support the development of New Rural Cooperative Medical System [10].

NCMS was also implemented with a clear policy guidance issued from central government. Before the implementation of NCMS, central government issued “Opinions on Establishing a New Rural Cooperative Medical System” in 2003, and this document included all principles for designing and implementing NCMS to guide lower level governments, including the lowest standard for premium level and subsidy level, fund management regulations, health services packages covered, and services quality management. In this technical guidance document, the roles of different actors involved in managing or supervising NCMS were also clearly defined [24].

### Systematic design

An effective health policy should build or adjust its health care delivery system and organizational structure in order to enable the implementation and fulfilling the aims of this policy; in other words, the system design should be fit for purpose. In addition, duplication and fragmentation should be avoided [4].

## CMS

The analysis of policy documents [26, 27] demonstrates that policy makers involved in the CMS were aware that CMS could not function well without corresponding accessible and well managed delivery system, thus the policy guidance document notes that CMS should make a full use of the three-tier health delivery system in rural areas and trained barefoot doctors, to provide services. The three-tier health providers were the only health providers in rural China during that period, and both the CMS and three-tier health delivery system were under the supervision and management of the government. Therefore, the CMS’s design and implementation did not involve arrangements on how CMS can select and contract with health providers.

The CMS design did not involve building new management departments, and in most areas the CMS fund was not managed by a separate department, but by the agricultural production cooperatives, who were also in charge of managing economic production and welfare issues in a village [31].

## NCMS

Compared with the CMS, the NCMS system design was specified in a more systematic manner, and an organizational structure was formed to support the NCMS fund management and policy operation. The management system includes three parts. The NCMS coordinating work team is composed of all relevant government departments (Health, Finance, Agriculture, and Civil Affairs), and its responsibility is to design and adjust the NCMS scheme. The NCMS management office is in charge of the operation of NCMS. Its management committee is composed of all relevant government departments and rural residents’ representatives, and the committee is in charge of supervision of NCMS performance and fund management [24]. To reduce the duplication and contain management costs, the NCMS management office is located within the structure of the health administration department.

There was also a recognition that NCMS could not operate effectively without strengthening of other health system components. The NCMS guidance document [23] included recommended action for further strengthening of the health delivery system in rural areas as an important policy component. One source also mentioned that in the process of NCMS expansion, the Ministry of Health made significant efforts to negotiate with Ministry of Finance and the National Development and Reform Commission, who are in charge of the country’s financial investment in primary health facilities and other aspects of health system strengthening [11]. In the national policy guidance document, there was a general directive that local government should choose health providers based on their performance [19], but with no specific advice on how to achieve this in practice. Contracting was also not common, and there were few contracts between health providers and NCMS in the early phase of the scheme. In recent years, with the improvement of the NCMS design, purchasing mechanisms seeking to influence the behaviour of health providers in terms of the quality and cost of health services that are provided, became more sophisticated, involving administrative reviews and appraisals of health providers seeking to provide certain services to NCMS patients, supervision of their performance, further development of the payment methods to include performance-related payment and formal contracting of providers [33, 34].

“*To strengthen the rural health delivery network, and strengthen the management of rural health care providers in order to improve the quality of health services provided to rural residents……. Local government need to select the designated health care providers for NCMS based on their performance, and should enhance the supervision on the health care providers. The guideline on diagnosis and treatment should be improved to enhance service quality, efficiency and control cost.*” (From policy document “Opinions on Establishing a New Rural Cooperative Medical System”, 2003) [19]

### Regulation and management capacity

In order to ensure the effective implementation of health policy, it is expected that a supportive governance system should introduce appropriate regulations and incentive mechanisms to influence the behaviour of government officials, managers and other relevant actors [4].

## CMS

**Fig. 1:**
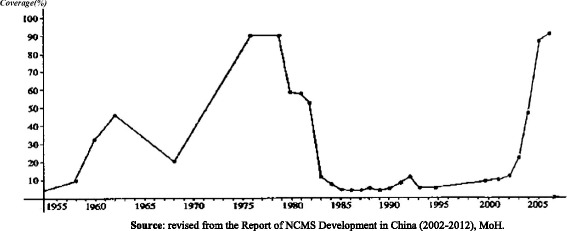
The percentage of villages covered by CMS and NCMS

The administrative system of China was highly hierarchical, and under this governance system policies were implemented in a top-down manner, with the higher administrative levels forwarding the regulations and policy implementation targets to the lower administrative levels. As Fig. 1 shows [35], the first sharp increase in coverage of CMS was at the end of the 1950s, and the most important regulations and rules were all issued during that period. The first regulation was issued in 1956, as noted above, the “Advanced Agricultural Production Cooperatives Demonstration Rules”. In November 1959, the national health work conference was held in Jishan County, Shanxi Province, during which the “Report on the National Health Work Conference in Jishan County” and its appendix “Opinions on People’s Communes Health Work” were presented and enacted [36].

The effectiveness of top-down regulations or targets on policy implementation was more pronounced if the document was endorsed at the higher level of decision making. For CMS, the strong political will by the highest leader, Mao Zedong, to promote the expansion of CMS was the most important driving force behind the rapid implementation of the newly enacted regulations and rapidly increasing coverage of CMS. In 1950s China just entered into peace after a long term war, the personal charisma of Chairman Mao Zedong was established in the long war history, and his advocating and emphasis on improving the health system was a very effective impetus for the implementation of health policies and particularly those related to the CMS. One month after the enactment of the above mentioned two guidance documents, in March 1960, Mao Zedong personally drafted “Instructions on Health Work” and emphasized that “*the Central Committee of the Communist Party dictates that the first Secretary of different levels of the communist party committees should lead CMS work and make sure the two documents are forwarded to each people’s commune*” [37]. Two years after this decree, the coverage of CMS showed its first cycle of rapid increase. The next round of promotion of CMS from the higher political echelon, demonstrating political will, began in 1968. One report analysing the experience of CMS at the Leyuan Commune, Hubei Province, was commended and advocated by Mao Zedong [10], which resulted in a second period of rapid increase of CMS coverage at the end of 1960s.

*“In the Zhangye District, Gansu Province, at that time, the CMS work was the first responsibility of the highest leader, the CMS work was emphasized at different kinds of meetings, and two large-scale health work meetings each year were held to discuss the CMS work.”(An interview cited from a literature)* [38]

Another way commonly applied to foster the CMS policy implementation in China was to strengthen the advocacy in relation to the scheme, channelled through the government media, whose viewpoints directly reflected the central government’s political strategy. In 1958, an article (“Introduction to the experience of CMS”) highlighting the importance of the scheme was published in Health Newspaper, run by the Ministry of Health [10]. From December 1968 to August 1976, People’s Daily, the Chinese Communist Party newspaper, published a column dedicated to discussing CMS in 107 issues [27], which reflected the sustained interest of high level policymakers in CMS and to keep in on the public radar.

Local governments’ capacity on design and management of CMS was also crucial for advancing the implementation of the CMS to the entire country. The practice of the Chinese government was to encourage local governments to learn from the experiences of other provinces and regions; the ability to identify and learn lessons was facilitated by the relatively uniform basic structures and organisational patterns. For example, following the experience of CMS of the Leyuan Commune, Hubei Province, was reported in the People’s Daily, more than 50,000 visitors from different provinces visited Leyuan Commune from 1968 to 1976 to gain understanding of the local CMS implementation model [39].

## NCMS

The establishment and development of the NCMS was included in the 1978 “Constitution of the People’s Republic of China” enacted by the Fifth National People’s Congress, in which the third chapter states that “*the government should develop social insurance, social welfare, free medical services, and cooperative medical schemes in order to ensure the labourers’ health right*” [10]. Then in 2003, the coverage of the NCMS in rural areas was also written into the new “Agriculture Law” [10]. “People’s Republic of China Social Insurance Act” was passed in 2011 and regulated that government should establish and improve the NCMS for rural residents.

According to the above laws stating the basic requirement for the development of NCMS, the NCMS continued to develop and being implemented in a top-down manner. In 2002, when the central government decided to promote NCMS as a priority policy, the first action was that China’s top leaders frequently discussed and referred to it in different events, emphasising that the government could do more in the area of rural health care. On October 19, 2002, the central government issued the “Decision on Further Strengthening of Rural Health Work” which explicitly mentioned that “*until 2010, the New Rural Cooperative Medical System will cover all rural residents; and in order to achieve this objective, governments would subsidize NCMS since 2003.*” [24] In order to formally launch the NCMS, on October 29 2002, the China National Rural Health Conference was held in Beijing. At this conference, the central government formally announced the establishment of NCMS as a major national policy to be supported in the near future. In accordance with the hierarchical administration system, all these decisions and actions by the central government serve to explicitly release the information to different levels of government and other relevant sectors (e.g.agriculture) that the implementation of NCMS should be a priority on their agenda. As a result, different levels of government responded to the request by the central government, and NCMS entered in a period of rapid and well documented expansion throughout China [40, 41].

In order to strengthen the implementation of NCMS, the coverage of the scheme became a performance indicator for NCMS managers, and achieving and maintaining specific coverage targets was key to increasing managers’ promotion prospects. For example, in Xinyuan County, the coverage target level was set at 80%. The responsibility to achieve and maintain this level lay with the lowest administrative level (village committee and township government) [42]. Therefore, the township governments and villagers’ committees were strongly encouraged and incentivized to mobilize the population for enrolment.

*“I could mobilize a team of 50 community workers and 80 village cadres to induce farmers and herders to join the NCMS and to ensure the enrolment percentage target.* (*An interview with an officer who was responsible for health work in one township government explained cited from a literature*) [41].

The earmarking of central transfers as matching funds based on enrolment rate was another important incentive for local governments to expand coverage. The public subsidies for NCMS come from central, provincial and local government. The subsidies of different levels of government were allocated in accordance with the number of individual participants, and the central government’s matching subsidies would only be transferred when the local government’s subsidies were in place. Under this mechanism, it was reported that there was no need to set specific targets for the enrolment rate of each province, however local governments would set they own contextually-relevant targets [40].

In order to strengthen local governments’ capacity for designing and implementing the NCMS, it was reported that an expert team was created to help guide counties’ NCMS design and pilots, to develop training materials and carry out training for local government officers and NCMS managers [11].

### Accountable and transparent

Good governance requires that all relevant actors are held publicly accountable, and transparency is required as a critical step of promoting accountability [4].

## CMS

In the CMS’s design, there were particular arrangements seeking to ensure that CMS managers were accountable to the local CMS enrolees. Firstly, during the implementation process, the central government never required a compulsory enforcement of the CMS nor that a unified design is implemented throughout the whole country. The central government encouraged the local authorities to adapt the CMS design to the local situation [27]. This arrangement sought to ensure that the design of the CMS was better suited to the local context and responsive to the needs of local residents.

Notably, some of the initial policy documents on the CMS design, providing more detailed regulation on fund management emphasised the need for local accountability. For example, in the “CMS Regulations of Masheng County”, it was stated that the “*CMS fund is managed in the appointed account by the commune credit cooperative, which is responsible for supervising how village clinics using the fund. Village clinics should report their fund use situation to CMS enrolees.”* [26] The same document also notes the requirement that meeting of representatives of the CMS enrolees should be held each year, and the participants had the right to check the financial situation of the CMS. However, there is a lack of documents illustrating how this kind of regulation was implemented in practice. According to one source, however, there were cases of CMS funds being diverted to other purposes or commune managers using their privilege to buy expensive medicines [43]. It is unclear if this was a frequent occurrence.

## NCMS

Similarly to the CMS, for the NCMS the central government only decreed the content of the policy and the principal requirements for its functioning, as a general strategic framework. The specific design on the key elements of the scheme, including the level of subsidy, the extent of the benefits package, co-payment level, were left to the discretion of the local governments [40]. The NCMS management committee at county level was composed of representatives from different government departments and rural residents’ representatives, and this committee directed and coordinated the operation of the NCMS [24]. Howwever, the NCMS differed from CMS in important ways. The NCMS had much stricter regulations with regard to the management and allocation of funds compared with the CMS. According to the regulation, the income and expenditures of the NCMS funds were made through an earmarked account, and there were unscheduled inspections of fund management by auditing or finance departments [24]. Given these systems in place, there was no reports on diverting NCMS funds to other purposes. The transparency of the NCMS fund income, expenditure, and reimbursement processes was also required by national and local management regulations. These practices were critical and contributed to the rapid expansion of the NCMS because the transparency and public information on the fund management and allocation improved the confidence of rural residents in the safety of their premiums. However, there was almost not information in the documents included in this analysis on how the NCMS acheived clear and transparent arrangements at the local level, what specific designs may have helped to improve accountability, for example on the appointment of health providers and the contents of the benefit package.

One paper analysed the features of the NCMS design which may have improved its accountability at the local level. Given the voluntary enrolment, participants were able to decide whether the system met their specific needs or not. Administrators, therefore, had to convince participants of the effectiveness and efficiency of the system and improve their motivation to enrol through ensuring a high quality service [39, 41]. Given the arrangement that the earmarked subsidies of different level governments being based on the actual enrolment rate, residents’ willingness to join and pay for the NCMS became vital for sustaining the funding of scheme. Therefore, voluntary enrolment combined with earmarked central transfers as matching funds formed a local feedback loop to improve the accountability of local NCMS managers to rural residents in their areas.

### Intelligence and oversight

A supportive governance system is expected to draw on intelligence and evidence generated throughout the process of policy formulation, implementation and a constant cycle of impact evaluation and redesign [4].

## CMS

The earliest design of the CMS was derived from the practice of grassroots communities and residents, but not based on evidence. The emergence of CMS was the result of the development of the Chinese cooperative economy [36, 44] with the government acting to build on locally socio-economic relationships.

“*The appearance of CMS was the natural. With the development of a collective economy, the agriculture cooperative started to input funds to village clinics; at the same time doctors and rural residents also started to input some funds into village clinics. In exchange, the rural residents could enjoy free consultation in the clinics.*” (an interview with Zhang Zikuan, a policy maker experiencing the development of CMS)

However, during the process of scheme development, the government sought to collect information, obtain and reflect on evidence on experiences of implementation across China. Carrying out field investigations in different geographical areas and learning from the practices piloted by local governments was a commonly used mechanism by the Chinese government to collect and use intelligence to inform policy making. CMS attracted the attention of high level policymakers in the short period after its establishment in 1955. The first step of the central government was to send the vice-minister of the Ministry of Health to visit Gaoping County and investigate CMS implementation. Even in the period from 1962 to 1968, when the development of CMS slowed down, the field investigations and proactive sharing of experience continued. In 1966, the vice minister of the Ministry of Health led a team to investigate the CMS in the Macheng Hubei Province where CMS had continued to develop. After a detailed investigation and participant observation, two reports analysed the implementation specificity of CMS in these areas and the accumulated evidence was disseminated to many actors and used effectively to inform the next round of rapid development of CMS [10]. At the same time, no evidence of interest in learning from international experience was found.

*“After two investigative visits, it was confirmed that CMS initially achieved early prevention of illness, early treatment after getting illness, low cost, and convenient utilization of health services, based on which the decision was made to promote the success of the CMS to the whole country.*(an interview with Zhang Zikuan, a policy maker experiencing the development of CMS) [31]

*“At that time, the field investigation was different from now. We lived in the home of farmers for two or three months, and observed the real situation on how CMS worked and its influences on peasants.”* (an interview with Zhang Zekuan, the team member of this time investigation)

## NCMS

The 1980s and 2000s saw proliferation of research, with many more studies on China’s rural health insurance and health inequities being undertaken. The research helped to accumulate the evidence base required for the initiation and continued refinement of the NCMS policy design. For example, a study supported by the Asian Development Bank (ADB) and co-sponsored by the State Development and Planning Commission (SDPC), and a policy briefing paper which introduced the findings brought to the attention of the to country Premier and Chairman that “*family bankruptcies due to medical expenses accounted for a third of the rural poverty.*” [23]. At the end of the 1980s, two studies, conducted by Anhui Medical University and Ministry of Health, compared CMS and the user fee-based model, and concluded that the CMS could help to improve health care unitization and health status compared with the user fee model [23, 45, 46]. The research studies achieved an impact throuogh placing the need for insurance on the policy agenda and demonstrating to the top leaders in China the need to rebuild China’s health insurance system in rural areas.

Other studies piloted and evaluated different policy designs. From 1986-1990, the project “Health insurance experiment in rural China” supported by the World Bank piloted alternatives to the CMS design, including premiums being 1-2% of household income, risk pooling at the township level, household enrolment, and main coverage of inpatient services, all of which directly contributed to the design of NCMS [47]. From 1992, the State Council China initiated a study on the feasibility of the re-introduction of the CMS, and in this study, the government piloted re-build of CMS in 14 counties in seven provinces [20]. The key contribution of the study, was that it piloted the government subsidy for premiums and concluded the necessity of government financial support for the new CMS. Another contribution of this project was that it encouraged more provinces to start their own pilots of new style of CMS. Since 1996, 19 provinces also started their CMS pilots involving research and evaluation. At the same period, there were several other projects which also confirmed that a new type of CMS was not feasible without government financial support. For example, in project “Strengthening basic health services in poor rural areas of China”, a Department for International Development (DFID) UK inputted fund to simulate the government subsidy [48].

In 2003, when the NCMS was formally launched by central government, pilots were also a crucial part of the implementation process. In the technical guidance document issued by the central government, there was no detailed design specifying the contents of scheme (premiums rate, provider payment mechanism, benefit package, etc.); but it was required that provinces need to select at least two to three counties to pilot their schemes prior to full-scale implementation. There was the expectation that the pilots will be evaluated, and any policy scale-up to all counties drawing on the evaluation outcomes. From 2003 to 2005, NCMS pilots were conducted in approximately 300 counties; and in 2006 a large-scale evaluation of already implemented NCMS was conducted [49]. All these pilots and evaluations helped to inform the final design of NCMS [10, 11].

“*The many efforts to re-establish a social health financing scheme in rural areas during the 1990s provided very useful lessons for policy makers.*” (an interview with the NCMS office director, cited in another study) [30]

### Collaboration and coalition building

Health system governance also involves process of coordination, collaboration and coalition building. Under a well-governed system, the government and non-state actors, working in the health sector and in other sectors relevant to health, are kept connected and jointly support the identifying of policy objectives, as well as generation and implementation of public policies addressing these [3].

## CMS

No information demonstrating collaboration between different departments during the process of CMS design and implementation was identified in the review.

## NCMS

The review found that the discordance among policies issued by different government departments may have impeded the rebuilding of the CMS in 1990s. Though Ministry of Health planned to collect premiums from rural residents for rebuilding the CMS, Ministry of Agriculture did not allow to add additional fees to the tax burden of the farmers [30].

In the early 2000s, given the high priority of rebuilding of NCMS on the national development agenda, the policies and actions of different government departments became increasingly more coordinated. In addition to the Ministry of Health, the Ministry of Civil Affairs also strongly supported the development of the NCMS [10], because Ministry of Civil Affairs was also aware of the acute problem of impoverishment caused by illness in rural China. In 2003, the key NCMS launch and guidance document “*Opinions on establishing a new rural cooperative medical system”* was jointly issued by the Ministry of Health, the Ministry of Agriculture, and the Ministry of Finance. Notably, the process of NCMS’ planning and implementation, a broader diversity of social actors, for example, academic researchers, took important roles in the process of NCMS design as discussed above.

“*In 1991, the Ministry of Health, Ministry of Agriculture, National Committee of Family Planning, National Committee of Education, and the Ministry of Personnel ever jointly issued a letter to the State Council “Asking for reforming and strengthening health work in rural areas”, in which they jointly asked for the re-building of health insurance schemes in rural areas.”(An case cited from a literature*) [10]

## Discussion

### Key findings: what does governance mean for strengthening the rural health insurance in China

This study reviewed and analysed the process of designing and implementing two insurance schemes in rural China, compared the governance practices underlying these processes, and their fit with the criteria for *good governance*. We identified a number of supportive governance practices common to the two schemes, including central government prioritising health system development; the specific health financing policies being also recognised as key within national development agenda; strong political will for promoting the policies drawing on the advantages of the highly hierarchical administrative system in China; local government autonomy in adoption of policy initiatives responding to local conditions but operating within the remit of a national policy framework; accumulating evidence generated from local experience to support policy making across China.

However, policy aspects and practices under some governance domains also suffered from considerable deficiencies. The national framework design for CMS was insufficiently comprehensive and systematic. Implementing CMS in some areas did not involve a corresponding adjustment in the management and organizational structures, and was managed by multi-purpose departments administering a heavy load of local programmes and initiatives. In contrast, the NCMS benefited by a newly established dedicated department to manage the funds, which ensured tighter management and accountability. Corruption and inefficient use of CMS funds was reported at places, with these occurrences less common under the NCMS. Importantly, collaboration between different government departments in the process of rural health insurance system development was often lacking and the discordance between policies introduced by different departments and sectors represented an obstacle to health insurance system strengthening in China.

The study demonstrated clearly how effective governance practices contributed to policy innovation and successful implementation. CMS and NCMS differed in terms of their origins, conceptualisation and initiation. CMS is a community-based health insurance stemming from China’s grassroots practices and collective economy, with policy makers in China becoming aware of its potential as a suitable policy option to reduce barriers to health care, and quickly seizing the opportunity to advocate and promote it. NCMS, in contrast, is a government-led scheme from its design and planning stage. However, both schemes were rapidly scaled up nationally. An important supportive factor common to both schemes was that the health system development was considered a priority on the country’s development agenda in the two time periods when the schemes were being institutionalised. The central government showed a substantial commitment to the development of the two policies, including political commitment for both schemes, and an added strong financial commitment for the NCMS. The high-level endorsement by key decision makers provided the basis for different departments designing flexible policies relevant to the local context, and was especially important for the rapid uptake of the insurance schemes across China. This signalling was particularly important in China where the political system is hierarchical and dominated by policy elites, and high level decision makers have considerable power and discretion with regard to setting strategic directions [23], investing in far-reaching initiatives with little consultation, and selecting local government officers with stake in implementation.

The issuing of decrees and regulations by the central government and assigning operational tasks to lower levels of governments were key mechanisms for implementing the CMS and NCMS nationally. However, the critical factors facilitating effective policy implementation were the strong commitment of the central government and the range of concrete and visible steps undertaken to ensure that this commitment is translated into action. For example, the directives issued by Mao Zedong played key role in expanding the coverage of the CMS, especially the requirement that the highest leader of local government should take charge of the CMS work, ensuring a local buy-in. The NCMS was also led by highest government officers at the different levels of government; at the same time, the effective roll-out of the NCMS and its coverage and management, became important performance indicators against which local governments were appraised. It could be argued that the presence of effective regulations and incentives to enforce the health policies and pursue faster implementation reflected the central government’s commitment and prioritisation of these policies, and this may have helped to build quicker the implementation capacity. This stewardship role for implementing large-scale initiatives may be more important in the more hierarchical health systems.

For both CMS and NCMS, the central government provided clear technical guidance, fundamental principles and requirements for the schemes’ design. However there was never a ‘one size fits all’ policy in China imposed on the implementers, which may be feature of policy development in countries with vast territory and multi-level administrative divisions. Seeking to facilitate the adoption of schemes aligned with the local context, the local governments were allowed considerable autonomy and encouraged modification of the policy design within the framework of the national requirements. The autonomy also functioned as an incentive for the local governments to actively pursue the implementation of new, potentially high-risk policies, since the well-performing pilots or successful policy uptake usually resulted in recognition and reward by the central government. Autonomy was accompanied by the above mentioned practice, i.e. scheme’s implementation and performance being set as an important indicator for assessing local government capability. This led to a feedback loop motivating the local governments to pursue policy innovation while at the same time contributing to national strategy development.

Another challenge in health system strengthening was the limited intelligence available to support policy design and adaptation of policies, for example the low interest to and uptake of lessons from other countries because of the limited linkages and supportive political context in China. Decision-makers sought to accumulate evidence from local practices implemented in different parts of the country including grassroots experiences with the CMS, generated through field visits and research, with their findings becoming the basis of influential policy guidance documents that were then disseminated nationally. The NCMS’s final policy design drew on a large number of studies and evaluations of policy pilots in many counties. This practice may have promoted the adoption of policy innovation and effective implementation in three ways: through the mobilization of intellectual resources at all administrative levels to design the policy; the central government’s recognition of local areas’ exploration and variations in the policy may have acted as an incentive for the local government to pursue a more appropriate policy design; and thirdly, the policy design was continuously refined considering that practices inside country would be more acceptable to different sectors and to different contexts, therefore accelerating and facilitating versatile implementation.

The analysis highlighted the importance of collaboration and coalition as markers of good governance in the policy process of roll-out CMS and NCMS in China. During the CMS period, there was no evidence that departments collaborated in the process of policy implementation; moreover, a lack of consensus among different departments impeded the rebuilding of CMS even over a relatively long period. Conversely, the initiating of NCMS was characterised by a higher level of collaboration which was achieved under the coordination of the central government and facilitated by a strong leadership by the Ministry of Health. Collaboration across different government departments has been a traditional bottleneck in China’s health system, because despite its hierarchical health system, national governance is still often fragmented, with the decision-making power dispersed in different governmental departments having powers in relation to key functions, including the technical support and supervision, financial support decision, and personnel management; with vertical lines of management and accountability [11]. In this system, the political interests differ among various interests groups and departments, and they must compete for political and economic resources [50]. This kind of adversarial national governance system is not conducive to easily reaching consensus and undertaking coordinated actions among departments and sectors, towards a common goal.

### Comparisons with other studies

There are a large number of studies on CMS and NCMS in China, and most have focused on policy design, coverage and their impacts on service accessibility and financial protection [9, 12, 13]. There are several studies analysing the policy process underlying the two schemes. Zhang [11] applied the concept of complex adaptive systems in analysing how rural health system developed given the rapidly changing context in China. Wang [10] analysed the development of the rural health care financing system as the process adopted by key policy makers, of continually learning from new practices and adopting to changed environments. Several other studies analysed specific aspects of the policy process, for example, how pilots of NCMS were conducted [20], how research impacted on policy making in NCMS [22], or how a district-designed and implemented local NCMS [40, 42]. Studies analysing overall health system in China also concluded that the institutional structures, political processes and procedures underpinning specific health policies may be more important as to why these policies were seen to work, rather than their design as such [51, 52], however these studies did not sufficiently explore the significance of governance for policy effectiveness.

This paper sought to address this gap by applying a dedicated governance and leadership framework to identify the specific governance factors and practices which supported the appropriate design and effective implementation of two health insurance schemes. The information search and extraction followed a rigorous search strategy and relied on a transparent screening process. We synthesized information on the design and implementation process of CMS and NCMS based on six domains of health system governance, with the broad concept of governance operationalised into specific sub-domains and questions, and these were used to structure and analyse the key emerging themes. In the process of matching of data and themes to the different governance domains, classifying the governance practices related to the CMS and NCMS policy formulation and implementation, to the specific governance functions was critical. During the analytical process, it became apparent that the same practices can be classified under different governance functions and subdomains. For example, Chair Mao Zedong’s personal influence on CMS’s implementation can be seen as an expression of strong policy will and pertaining to ‘policy vision and guidance’; while it can also be relevant to ‘regulation’ because his endorsement of the policy, from the position of a top leader, may have accelerated the implementation of regulations enacted at national level. These discrepancies were discussed within the team, and in collaboration with senior researchers in China, and the final classification was cross-examined through regular contact with the international project experts with experience of governance analysis in other settings. The findings of the review were also validated by senior researchers and policy makers who were involved in the implementation process of CMS or NCMS.

### Policy implications

The political move towards universal coverage as a major health system goal has led many low- and middle-income countries to initiate reform or improve their health financing system to expand the breadth and depth of population coverage. Expanding health insurance coverage to rural areas or the informal sector has often been an important element of such strategies. China established the CMS and NCMS in different historical stages, and both schemes contributed to an improved access to health services, reduction of disease and alleviation of the financial burden for rural residents. Crucially, CMS and NCMS are seen internationally as examples of policy innovations that were rapidly scaled up to achieve near universal coverage. At the same time, a number of low-income countries have faced considerable challenges in achieving major improvements in coverage and extending basic financial protection to their disadvantaged populations despite long term efforts [53, 54]. This study demonstrated that health systems governance may be a critical underpinning mechanism that enables policy innovation, strategic development appropriate to sub-national contexts, and effective implementation through a consistent policy cycle of piloting, implementing on a large scale, learning lessons and readjusting policies.

Our analysis suggests that large-scale initiatives to expand health insurance coverage to rural, informal and marginalised population should understand and address not only constraints related to financing arrangements and organisational capacity but also identify what good practices need to be enacted in each governance domain to support the development of such schemes. As discussed above, the experience of China in implementing the CMS and NCMS may provide a range of useful lessons. At the policy design stage, encouraging sub-national governments to pilot diverse policy options, accumulate evidence and disseminate experiences from local practice, and compare these with areas with different characteristics, could help to test and identify a range of policy options which fit with local health systems, and are feasible and acceptable to different stakeholder, including those beyond the health system. Clear accountability lines and relationships can help to synthesise local experience and ensure it shapes strategies owned at national level. At the stage of scaling up schemes nationally, leadership by national institutions through enacting regulations and setting policy goals and targets to sub-national government authorities and assessing policy implementation as evaluation of local government performance can offer leverage. A caveat is that this maybe more applicable to systems that tend to be more hierarchical, although it should be noted that China combines centralised decision making with considerable autonomy at the province level.

The critical point is that the central government should explicitly define the priority of key health policies, in this case health insurance schemes for the rural populations, within the overall national development agenda which is usually contested by multiple actors in LMICs. Achieving this is the basis for effective planning and implementation of large-scale policy initiatives to expand health care coverage. Many of the features of the Chinese health financing reform are compatible with the definitions of good governance referred to in this paper. Governance appears to be a cross-cutting building block enabling developments across other areas of the health system. There are indications that fostering good governance through attention to policies and actions in relation to all its domains, can enable LMICs implementing ambitious government-led financing initiatives to accelerate progress.

## Conclusions

China’s success in achieving scale up of CMS and NCMS has attracted considerable interest in many low and middle income countries, especially with regard to the schemes’ designs, coverage, and their impacts on services accessibility and financial protection. However, this study demonstrates that health systems governance may be critical to enable the planning, design and implementation of such schemes. Given that many LMICs are expanding health financing system to cover populations in rural areas or the informal sectors, we argue that strengthening specific practices in each governance domain could inform the adaptation of China experiences in rural health insurance system strengthening to other settings.

## Abbreviations

ADB: Asian development bank
CMS: Cooperative medical scheme
CNKI: China national knowledge infrastructure
DFID: Department for International development
LMIC: Low and middle income countries
NCMS: New rural cooperative medical scheme
SDPC: State development and planning commission

## References

[1] Siddiqi S, Masud TI, Nishtar S, Peters DH, Sabri B, Bile KM, Jama MA (2009). Framework for assessing governance of the health system in developing countries: gateway to good governance. Health Policy.

[2] Brinkerhoff D, Fort C, Stratton S. Good governance and health: assessing progress in Rwanda. Twubakane. Decentralization and Health Program Rwanda. USAID. 2009. http://www.intrahealth.org/~intrahea/files/media/good-governance-and-healthassessing-progress-in-rwanda/goodgovandhealth.pdf. Accessed 28 Jun 2016.

[3] Balabanova D, Mills A, Conteh L, Akkazieva B, Banteyerga H, Dash U (2013). Good health at low cost 25 years on: lessons for the future of health systems strengthening. Lancet.

[4] World Health Organization. Everybody business: strengthening health systems to improve health outcomes: WHO’s framework for action. Geneva 2007. http://www.who.int/healthsystems/strategy/everybodys_business.pdf. Accessed 28 Jun 2016.

[5] Travis P, Egger D, Davies P, Mechbal A (2001). Towards better stewardship: concepts and critical issues.

[6] Dodgson R, Lee K, Drager N. Discussion paper no. 1: global health governance; a conceptual review. Centre on Global Change & Health, Department of Health & Development London School of Hygiene and Tropical Medicine and World Health Organization, 2002.

[7] Joshi A, Moore M (2004). Institutionalised co-production: unorthodox public service delivery in challenging environments. J Dev Stud.

[8] Meng Q, Xu K (2014). Progress and challenges of the rural cooperative medical scheme in China. Bull World Health Organ.

[9] Meng Q, Xu L, Zhang Y, Qian J, Cai M, Xin Y (2012). Trends in access to health services and financial protection in China between 2003 and 2011: a cross-sectional study. Lancet.

[10] Wang S (2008). Learning and adapting: The case of rural healthcare financing in China. Soc Sci China.

[11] Zhang X, Bloom G, Xu X, Chen L, Liang X, Wolcott SJ (2014). Advancing the application of systems thinking in health: managing rural China health system development in complex and dynamic contexts. Health Res Policy Syst.

[12] You X, Kobayashi Y (2009). The New Cooperative Medical Scheme in China. Health Policy.

[13] Liang X, Guo H, Jin C, Peng X, Zhang X (2012). The effect of New Cooperative Medical Scheme on health outcomes and alleviating catastrophic health expenditure in China: a systematic review. PLoS ONE.

[14] United Nations Development Programme. Governance for sustainable human development: a UNDP policy document. New York: UNDP; 1997. http://www.pogar.org/publications/other/undp/governance/undppolicydoc97-e.pdf. Accessed 28 Jun 2016.

[15] Barnett-Page E, Thomas J (2009). Methods for the synthesis of qualitative research: a critical review. BMC Med Res Methodol.

[16] Glenton C, Colvin CJ, Carlsen B, Swartz A, Lewin S, Noyes J, et al. Barriers and facilitators to the implementation of lay health worker programmes to improve access to maternal and child health: qualitative evidence synthesis. Cochrane Database Syst Rev. 2013; doi: 10.1002/14651858.CD010414.pub2. 10.1002/14651858.CD010414.pub2 PMC6396344 24101553

[17] Zhang Z, Zhu Z, Wang S, Zhang C (1994). Retrospective study on rural health care insurance schemes in China. Chin Rural Health Serv Adm.

[18] Zhou S (2002). Exploring the development trend of rural health insurance schemes. Int Med Health Guid News.

[19] Zhu L (2000). The government and options for Alternative Medical and Health Systems in rural areas. Soc Sci China.

[20] Carrin G, Ron A, Hui Y, Hong W, Tuohong Z, Licheng Z (1999). The reform of the Rural Cooperative Medical System in the People’s Republic of China: interim experience in 14 pilot counties. Soc Sci Med.

[21] Zhang D (2005). Review and reflection on development of rural medical and health care in China. Health Econ Res.

[22] World Health Organization. The world health report 2000 - Health systems: improving performance. Geneva, Switzerland: the World Health Organization; 2000.

[23] Liu Y, Rao K (2006). Providing health insurance in rural China: from research to policy. J Health Policy Law.

[24] Ministry of Health. Collections NCMS Policy Documents (2002–2011). Beijing: Department of Rural Health Management, MoH; 2012.

[25] Meng Q, Fang H, Liu X, Yuan B, Xu J (2015). Consolidating the social health insurance schemes in China: towards an equitable and efficient health system. Lancet.

[26] Zhang Z. Temporary management methods for cooperative medical system in Masheng County (Draft). In “Experiencing Rural Health Work for Sixty Years – Anthology of Zhang Zikuan on rural health”. Beijing: China Union Medical College Press. 2011. p. 300-303.

[27] Zhang Z. Actively implementing the cooperative health care system for commune member. Health News (Jian Kang Bao). 1960 May 18.

[28] Yip W, Powell-Jackson T, Chen W, Hu M, Fe E, Hu M, Jian W, Lu M, Han W, Hsiao WC (2014). Capitation combined with pay-for-performance improves antibiotic prescribing practices in rural China. Health Aff (Millwood).

[29] Jing L, Bai J, Sun X, Zakus D, Lou J, Li M, Zhang Q, Zhuang Y (2016). NRCMS capitation reform and effect evaluation in Pudong New Area of Shanghai. Int J Health Plann Manage.

[30] Cao P (2006). The Rural Cooperative Medical System in the People’s Republic of China before 1978. Documents Communist Party China.

[31] Zhang ZK (1992). Development of the cooperative medical system in China:a review of history. Chin Health Econ.

[32] Bogg L, Dong H, Wang K, Cai W, Vinod D (1996). The cost of coverage: rural health insurance in China. Health Policy Plan.

[33] Wu Q (2009). Research on contractual regulation of grassroots medical organization: From the angle of relational contract theory. Master Thesis.

[34] Huang L (2011). The study of supervision mechanism for certified medical institutions of the New Rural Cooperative Medical Scheme. Master Thesis.

[35] Chen Z, Zhang M (2013). China New Rural Cooperative Scheme development report, 2002-2012.

[36] Yue QH HEPY (2007). Critical review on the development of public health care from 1949-1984, in Ji Shan County, Shan Xi Province: taking Sun village as a key object of investigation. Contemp China Hist Stud.

[37] Mao ZD (1996). Instructions of the health work from the Communist Party of China. Chairman Mao Zedong’s manuscripts since 1949.

[38] Wang M (2006). Institutional embeddedness and keep-on development of the cooperative medical schemes (CMSs) operating in rural Chin.

[39] Hu ZD (2006). The unsung hero and his career of taking the Rural Cooperative Medical System into the whole country. Employ Secur.

[40] Klotzbücher S, Lässig P (2009). Transformative State Capacity in Post-Collective China: The introduction of the New Rural Cooperative Medical System in two counties of Western China, 2006–2008. Eur J East Asian Stud.

[41] Zhang ZK, Zhu ZH, Wang SC, Zhang CY (1994). A review of the history of the rural cooperative medical system in China. Chin Rural Health Serv Adm.

[42] Klotzbücher S, Lässig P (2010). What is New in the New Rural Co-operative Medical System? An Assessment in One Kazak County of the Xinjiang Uyghur Autonomous Region. China Q.

[43] To strengthen the management on cooperative medical system (2011). Talk by Zhang Zikuan at Mansheng County. 13 October 1965. “Experiencing Rural Health Work for Sixty Years – Anthology of Zhang Zikuan on rural health”.

[44] Wang JY, Xu DB (2005). The history of the Cooperative Medical System and practices of the New Rural Cooperative MedicalSystem in Ganyu County. Jiangsu Health Care.

[45] Qian WY (2006). Historical development of the Rural Cooperative Medical System from 1949-1979, in Zhe Jiang province, China. J Anhui Agric Univ.

[46] Zhu A (1988). A series of study on the Rural Cooperative Medical System in China. Chin Health Econ.

[47] Cretin S, Williams AP, Sine J. China Rural Health Insurance Experiment: final report. RAND Health Working Papers WR-411. 2007. http://www.rand.org/pubs/working_papers/WR411.html. Accessed 28 Feb 2017.

[48] Wang SD, Ye YD (2004). Study on the Rural Cooperative Medical System: history and future. Chin Primary Health Care.

[49] Lin C, de Haan A, Zhang X, Warmerdam W (2011). Addressing vulnerability in an emerging economy: China’s New Cooperative Medical Scheme (NCMS). Can J Develop Stud.

[50] Wang SG (2007). From economic policy to social policy: The historic transformation of China’s public policy structure. China’s public policy review.

[51] Zhang W, Li C, Bloom G (2011). Building institutions for an effective health system: lessons from China’s experience with rural health reform. Soc Sci Med.

[52] Peters D, El-Saharty S, Siadant B, Janovsky K, Vujicic M (2009). Improving health service delivery in developing countries: from evidence to action.

[53] Gemini M, Jo-Ann M. Community Health Funds in Tanzania: A literature review. The Consortium for Research on Equitable Health Systems (CREHS). January 2007. http://www.crehs.lshtm.ac.uk/downloads/publications/Community%20health%20funds%20in%20Tanzania.pdf Accessed 28 Jun 2016.

[54] World Health Organization. The world health report 2010 - Health systems financing: the path to universal coverage. Geneva, Switzerland: the World Health Organization; 2010. 10.2471/BLT.10.078741 PMC2878164 20539847

